# Protective Effects of a Novel Probiotic *Bifidobacterium pseudolongum* on the Intestinal Barrier of Colitis Mice via Modulating the Pparγ/STAT3 Pathway and Intestinal Microbiota

**DOI:** 10.3390/foods11111551

**Published:** 2022-05-25

**Authors:** Weiling Guo, Bingyong Mao, Shumao Cui, Xin Tang, Qiuxiang Zhang, Jianxin Zhao, Hao Zhang

**Affiliations:** 1 State Key Laboratory of Food Science and Technology, Jiangnan University, Wuxi 214122, China; 7200112062@stu.jiangnan.edu.cn (W.G.); maobingyong@jiangnan.edu.cn (B.M.); cuishumao@jiangnan.edu.cn (S.C.); xintang@jiangnan.edu.cn (X.T.); zhangqx@jiangnan.edu.cn (Q.Z.); zhaojianxin@jiangnan.edu.cn (J.Z.); 2 School of Food Science and Technology, Jiangnan University, Wuxi 214122, China; 3 Ningbo Yuyi Biotechnology Co., Ltd., Ningbo 315153, China; 4 National Engineering Research Center for Functional Food, Jiangnan University, Wuxi 214122, China

**Keywords:** *Bifidobacterium pseudolongum*, intestinal barrier, PPARγ/STAT3 pathway, intestinal microbiota

## Abstract

Colitis has become a major health concern worldwide. The objective of the present study was to determine the probiotic influence of different strains of *B. pseudolongum* (Bp7 and Bp8) on alleviating colitis and to explore its possible potential mechanisms. Our results displayed that Bp7 and Bp8 intervention effectively reduced dextran sodium sulfate (DSS)-caused body weight loss and the release of several pro-inflammatory factors (interleukin (IL)-6, IL-1β, and tumor necrosis factor-α (TNF-α)) and increased the activities of antioxidant enzymes (T-AOC, SOD, and GSH) and the concentrations of tight junction proteins (occludin, claudin-1, and ZO-1). Moreover, Bp7 and Bp8 intervention drastically down-regulated the expression of colonic MyD88, NF-κB, iNOS and COX2 and drastically elevated the expression of colonic STAT3, Nrf2, and PPARγ. Gas chromatography-mass spectrometry results revealed that the cecal levels of isobutyric, butyric, and isovaleric acids were drastically increased in colitis mice intervened with Bp7 and Bp8. Moreover, 16S rRNA sequencing revealed that Bp7 and Bp8 intervention modulated the intestinal microbiota structure, particularly by enhancing the proportion of *Eubacterium coprostanoligenes group*, *Marvinbryantia*, *Enterorhabdus*, *Faecalibaculum*, *Coriobacteriaceae* UCG 002, *Alistipes,* and *Bifidobacterium*, which are relevant to the levels of cecal isobutyric acid, butyric acid, isovaleric acid, and inflammatory cytokines. Collectively, these findings suggest that Bp7 and Bp8 intervention alleviates the intestinal barrier function, possibly by blocking the secretion of proinflammatory cytokines, maintaining the intestinal physical barrier integrity, activating the PPARγ/STAT3 pathway, and modulating intestinal microbiota composition. Our study also suggested that *B. pseudolongum* is a promising probiotic for colitis treatment.

## 1. Introduction

Inflammatory bowel diseases (IBDs), comprising Crohn’s disease (CD) and ulcerative colitis (UC), are chronic refractory inflammatory diseases of the gastrointestinal tract. According to a previous investigation, IBD affects millions of individuals, and the global prevalence and occurrence of IBD are rapidly rising [1]. Multiple studies have suggested that IBD is significantly associated with excessive inflammatory cytokines, intestinal barrier damage, and abnormal intestinal microbiota. Among those, the intestinal barrier is mainly made up of intestinal epithelial cells (IECs) that not only prevent microbial invasion but also regulate mucosal homeostasis [2]. In addition, IECs participate in regulating inflammatory responses by producing antimicrobial peptides [3]. Conversely, some evidence has confirmed that the destruction of the intestinal barrier causes severe tissue damage and microbial invasion [4]. Therefore, the protection and restoration of the intestinal barrier are critical in preventing the invasion of intestinal microbes into the gut tissue and decreasing the inflammatory response. A previous study suggested that the PPARγ/STAT3 pathway plays an anti-inflammatory role at the intestinal barrier [5]. The activation of the PPARγ/STAT3 pathway prevents the secretion of pro-inflammatory cytokines and promotes the production of anti-inflammatory cytokines by monitoring the NF-κB pathway [5]. Thus, the activation of the PPARγ/STAT3 pathway is widely regarded as a potential therapeutic strategy for the repair of the intestinal barrier function.

The intestinal microbiota consists of trillions of commensal microbes, which play a vital role in maintaining the intestinal barrier function. A clinical study demonstrated marked differences in the intestinal microbiota structure between patients with IBD and healthy individuals, especially with regard to microbial diversity and the proportion of specific bacterial taxa [6]. For example, the diversity of intestinal microbiota in IBD patients was obviously decreased compared with that in healthy individuals [7]. The proportion of harmful bacteria (*Escherichia*, *Streptococcus,* and *Veillonella*) is commonly increased and the relative abundance of beneficial bacteria (*Bacteroides*, *Flavobacterium*, and *Oscillospira*) is reduced in patients with IBD [8]. Increases in harmful bacteria elevate the probability of direct interaction between the microbes and host tissue, thereby accelerating the occurrence and development of IBD. Moreover, recent reports have shown that an imbalance of the intestinal microbiota not only destroys the intestinal barrier via increasing the expression of MyD88 but also promotes the release of pro-inflammatory cytokines via activating the NF-κB pathway [9]. Hence, maintaining a healthy balance of intestinal microbiota is important for preventing or/and treating IBD.

At present, antibiotics, aminosalicylates, corticosteroids, and immunosuppressants are the most universal treatment agents for IBD. However, clinical evidence suggests that long-term intake of these drugs induces a series of adverse effects, for instance, allergic reactions, metabolic disorders, and liver diseases [10]. Consequently, it is imperative to search for more effective and less toxic natural bioactive substances or probiotics to avoid these side effects and to effectively treat IBD. A previous study exhibited that *Lactobacillus helveticus* and *L. rhamnosus* could attenuate colitis by decreasing the levels of pro-inflammatory cytokines, shifting the intestinal microbiota community structure and regulating the activities of antioxidant enzymes [11]. For example, *Lactiplantibacillus plantarum* AR113 ameliorates colitis symptoms by recovering goblet cell counts and tight junction proteins, reducing pro-inflammatory cytokines and mitigating intestinal microbiota dysbiosis [12]. In addition, an earlier study revealed that *Bifidobacterium adolescentis* ameliorates colitis via decreasing pro-inflammatory cytokines, regulating Treg/Th2 response, and reshaping intestinal microbiota composition [13]. However, despite being one of the most important members of *Bifidobacterium* spp., *Bifidobacterium pseudolongum* has not been studied in detail to explore its role in the treatment of colitis.

In this study, the ameliorating influence of *B. pseudolongum* (Bp7 and Bp8) on intestinal barrier damage in colitis mice was explored via detecting the levels of inflammation, oxidative stress, and tight junction protein. Moreover, quantitative RT-PCR of colonic genes and high-throughput sequencing of intestinal microbiota were undertaken to explore the potential mechanism of *B. pseudolongum* (Bp7 and Bp8) on ameliorating intestinal barrier damage. The relationships between inflammatory cytokines and intestinal microbial phylotypes was also determined out via correlation analysis. Overall, the aim of this study was to supply a theoretical basis for developing and utilizing novel *Bifidobacterium* to improve injury to the intestinal barrier function.

## 2. Materials and Methods

### 2.1. Preparation of Bacterial Suspension

*B. pseudolongum* strains (Bp7 and Bp8) were obtained from chicken manure, and their numbers were FYNLJ90M1 and FSCPS86M2, respectively. *B. pseudolongum* was cultured in De Man, Rogosa, and Sharpe (MRS) medium at 37 °C in an anaerobic workstation, and then 2% of the culture was inoculated into a new sterile MRS medium. Subsequently, the solution was centrifuged (12,000× *g*, 5 min), and the strains were rinsed six times with PBS. Finally, the collected strains were dissolved in skim milk (13% *w*/*v*), and the concentration was 5 × 10^9^ CFU/mL.

### 2.2. Animal Experiments

Thirty-two 7-week-old male SPF C57BL/6J mice (19–21 g) were procured from Jiangsu Laboratory Animal Center (Nantong, China) and housed under a standardized environment (temperature: 21–24 °C; humidity: 56–65%; and 12 h light/dark cycle). After 7 days of adaptation, all mice were equally divided into 4 groups. The control (NC) and model (DSS) groups were fed with 0.2 mL of skim milk (13% *w*/*v*) every day. The Bp7 and Bp8 groups were fed with 0.2 mL of skim milk containing Bp7 and Bp8 (1 × 10^9^ CFU/day), respectively (Figure S1). The mice in the NC group were provided with sufficient water during the entire process of the experiment. The mice in the DSS, Bp7, and Bp8 groups were provided with sufficient water for one week; subsequently, they were provided drinking water containing 3% (*w*/*v*) dextran sodium sulfate (DSS) from day 8 to day 14. The body weight was recorded daily. The whole experiment was approved by the Ethics Committee of Jiangnan University (JN.No20210430c1500608[097]).

### 2.3. Measurement of Disease Activity Index

During the animal experiment, the disease activity index (DAI) was measured every day according to a previous study [13]. Briefly, DAI scores consisted of weight loss score, stool consistency score, and stool bleeding score (Table S1). The score information is shown in Table S1. The DAI was computed according to the following formula:

DAI = weight loss score + stool consistency score + stool bleeding score

### 2.4. Measurement of Cell Cytokines and Antioxidant System in the Colon

Colonic TNF-α, IL-1β, IL-6, IL-10, IL-22, and mucin2 (Muc2) levels were measured using commercial kits (R&D Systems Co., Ltd., Emeryville, CA, USA) following the manufacturer’s instructions. The activities of myeloperoxidease (MPO), malondialdehyde (MDA), superoxide dismutase (SOD), and glutathione (GSH) were analyzed using commercial kits (Elabscience, Wuhan, China).

### 2.5. Histological Analysis

Histopathological examination of colon tissues was carried out as reported previously [9]. Briefly, the colons were removed, fixed, and embedded in paraffin. Hematoxylin-eosin staining, alcian blue staining, periodic acid-schiff (PAS) staining, and histological scoring were performed. Three randomly selected areas were observed per slide. The histological injury score was calculated as the average of each individual score.

### 2.6. Immunohistochemical Analysis

Immunohistochemical analysis was carried out following our previous study [9]. Briefly, the sections of the colon were incubated with primary antibodies specific to occludin (Abcam, ab216327), claudin-1 (Abcam, ab180158), and ZO-1 (Abcam, ab221546) for 60 min, followed by incubation with secondary antibody and streptavidin-biotin peroxidase for 120 min. Ultimately, the brown color was measured under 3,3′-diaminobenzidine tetrahydrochloride using a light microscope. The density of occludin, claudin-1, and ZO-1 were measured in three different areas of the colon tissues from the three groups using Image J 1.8.0.

### 2.7. Quantitative RT-PCR

Total RNA was obtained from the colon using a commercial kit (Jisai, Guangzhou, China) and reverse-transcribed into complementary DNA (cDNA) using a commercial kit (Vazyme, Nanjing, China). The mRNA transcription levels were estimated using an RT-qPCR system (Bio-Rad, Emeryville, CA, USA). The sequences of the primers used in this study are listed in Table S2.

### 2.8. SCFA Analysis

Cecal SCFA concentrations were obtained following a method from a previous study [14]. Gas chromatography–mass spectrometry was applied to measure the cecal SCFA concentrations (30–60 mg dry weight).

### 2.9. Bioinformatics Analysis of 16S rRNA Gene Sequences

Genomic DNA was obtained from frozen fecal samples using a DNA isolation kit (Xindalu, Fuzhou, China) following the manufacturer’s instructions. The 16S rRNA genes (V3–V4 region) were amplified using forward (338F) and reverse (806R) primers. The PCR products were purified using a commercial kit (Xindalu, Fuzhou, China), quantified using a NanoDrop ND-1000 spectrophotometer (Thermo, Waltham, MA, USA), and sequenced using an Illumina MiSeq sequencer at Jiangnan University (Wuxi, China).

The raw data from high-throughput sequencing were filtered using the QIIME software package (v 1.8.0). The results were annotated to operational taxonomic units (OTUs) when the value of similarity was greater than or equal to 97%. Principal coordinates analysis (PCA) scores and hierarchical clustering analysis (HCA) were implemented using SIMCA (v 14.1.0). STAMP (v 2.1.3) software was applied to identify intestinal microbial phylotypes with obvious difference among the groups. The metabolic pathways of intestinal microbiota were performed using PICRUSt2. The predictive associations were explored by Pearson correlation coefficients and the network was visualized by R software and Cytoscape v 3.6.1, respectively.

### 2.10. Statistical Analysis

To calculate the significance of the differences between groups, the data were analyzed using one-way ANOVA with GraphPad Prism (Ver. 7.0) software, followed by Tuckey’s multiple comparison test. In addition, *p* < 0.05 was considered statistically significant.

## 3. Results

### 3.1. Influence of B. pseudolongum Intervention on Body Weight and Colon Morphology in Colitis Mice

**Figure 1:**
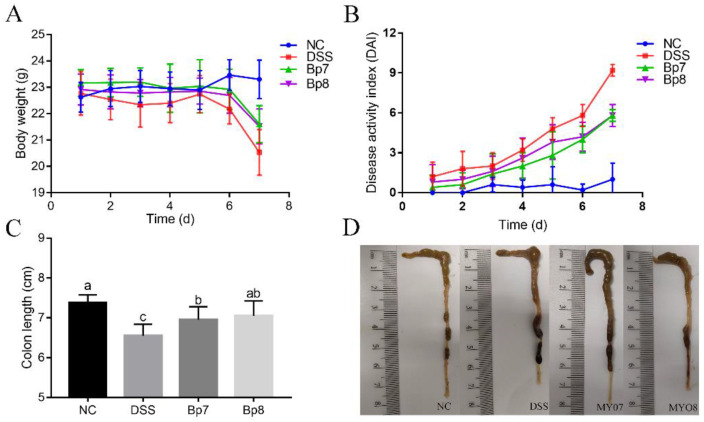
Influence of Bp7 and Bp8 intervention on body weight (**A**), disease activity index (DAI) (**B**), colon length (**C**), and representative pictures of colons in colitis mice (**D**) (*n* = 8). The different letters represent significant differences between different experimental groups (*p* < 0.05).

Relative to the NC group, the body weight was drastically reduced in colitis mice. However, the oral administration of Bp7 and Bp8 inhibited body weight loss (Figure 1). The DAI score was widely applied to assess the severity of colitis in individual mice. The DAI score in the DSS group was remarkably enhanced compared with that in the NC group (*p* < 0.05), whereas Bp7 or Bp8 intervention resulted in a sharp reduction in DAI score (45.35% and 42.67%, respectively). The colon length notably shortened in the DSS group (7.05 ± 0.9 cm) relative to that in the NC group (7.88 ± 0.20 cm). Compared with the DSS group, colitis mice treated with Bp7 or Bp8 partially reversed the effect of DSS treatment (7.45 ± 0.32 and 7.55 ± 0.37 cm, respectively).

### 3.2. B. pseudolongum Intervention Relieves Colonic Cell Cytokines and Oxidative Stress in Colitis Mice

**Figure 2:**
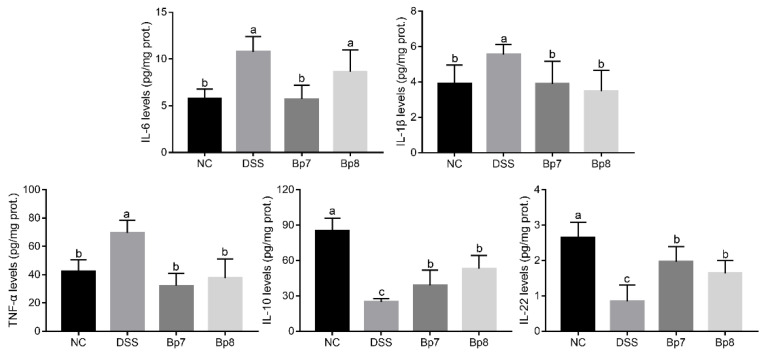
Influences of Bp7 and Bp8 intervention on the colonic inflammatory cytokines (*n* = 8). The different letters represent significant differences between experimental groups (*p* < 0.05).

As shown in Figure 2, the concentrations of colonic IL-6, IL-1β, and TNF-α significantly increased and the levels of colonic IL-10 and IL-22 levels were remarkably reduced in the DSS group relative to the NC group (*p* < 0.05). As expected, colitis mice treated with Bp7 and Bp8 exhibited lower concentrations of TNF-α and IL-1β (*p* < 0.05) and higher concentrations of IL-10 and IL-22 relative to the DSS group (*p* < 0.05). Moreover, Bp7 intervention significantly reduced the colonic IL-6 levels in mice with DSS treatment (*p* < 0.05).

**Figure 3:**
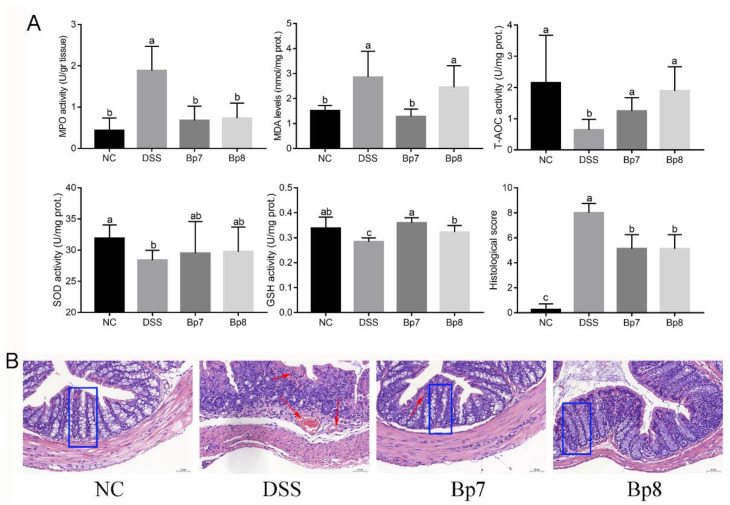
Influence of Bp7 and Bp8 intervention on the activity of antioxidant-related parameters and the histological injury in the colon. The activities of MPO, MDA, T-AOC, SOD, GSH and histological score (*n* = 8) (**A**); Histological analysis of colon tissue (the red arrows show inflammatory cell infiltration; blue frame show crypts) (**B**). The different letters represent significant differences between experimental groups (*p* < 0.05).

To further explore the ameliorative effects of Bp7 and Bp8 on intestinal barrier damage, the levels of colonic oxidative stress were analyzed. DSS treatment drastically increased the MPO activity and MDA levels in the mice of the DSS group relative to those in the NC group (*p* < 0.05) (Figure 3A). Interestingly, MPO activity and MDA levels were drastically decreased after Bp8 intervention and especially Bp7 intervention (*p* < 0.05). Moreover, the activities of T-AOC, SOD, and GSH were remarkably decreased in the DSS group relative to those in the NC group (*p* < 0.05), whereas Bp7 and Bp8 intervention significantly elevated the activities of T-AOC and GSH (*p* < 0.05). Nevertheless, there was no remarkable discrepancy in SOD activity among all mice with DSS treatment (*p* > 0.05).

The results of the colon tissue staining are shown in Figure 3B. The image of the colon from the NC group showed normal mucosal architecture. However, DSS treatment resulted in obvious histological alterations, including increased inflammatory cell infiltration, loss of crypts, decrease in goblet cells, and thickening of the muscular mucosae. However, these pathological changes were attenuated by Bp7 and Bp8 intervention. Based on these results, *B. pseudolongum* (Bp7 and Bp8) attenuated DSS-induced histopathological changes in the colon.

### 3.3. B. pseudolongum Intervention Attenuates Gut Barrier Integrity in Colitis Mice

**Figure 4:**
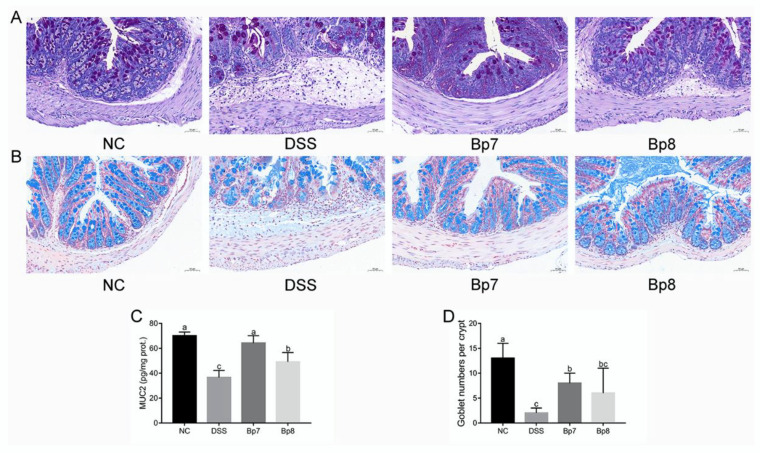
Influence of Bp7 and Bp8 intervention on the foundation barrier. Alcuin blue staining of colon (**A**); PAS staining of colon (**B**); Muc2 concentration in the colon (*n* = 8) (**C**); Goblet cell number in the colon (*n* = 8) (**D**). The different letters represent significant differences between experimental groups (*p* < 0.05).

The colonic mucus layer is regarded as the foundation barrier against the invasion of bacteria and toxins and prevents the development of colitis. Alcian blue staining and PAS staining showed that the colonic mucus layer and goblet cells were obviously destroyed after DSS treatment (Figure 4A,B). In addition, remarkably higher levels of Muc2 were observed in Bp7 and Bp8 groups relative to those in the DSS group (*p* < 0.05) (Figure 4C). Furthermore, Bp7 intervention remarkably elevated the goblet cell number relative to that in the DSS group (*p* < 0.05), but Bp8 intervention did not affect the number of goblet cells (*p* > 0.05) (Figure 4D).

**Figure 5:**
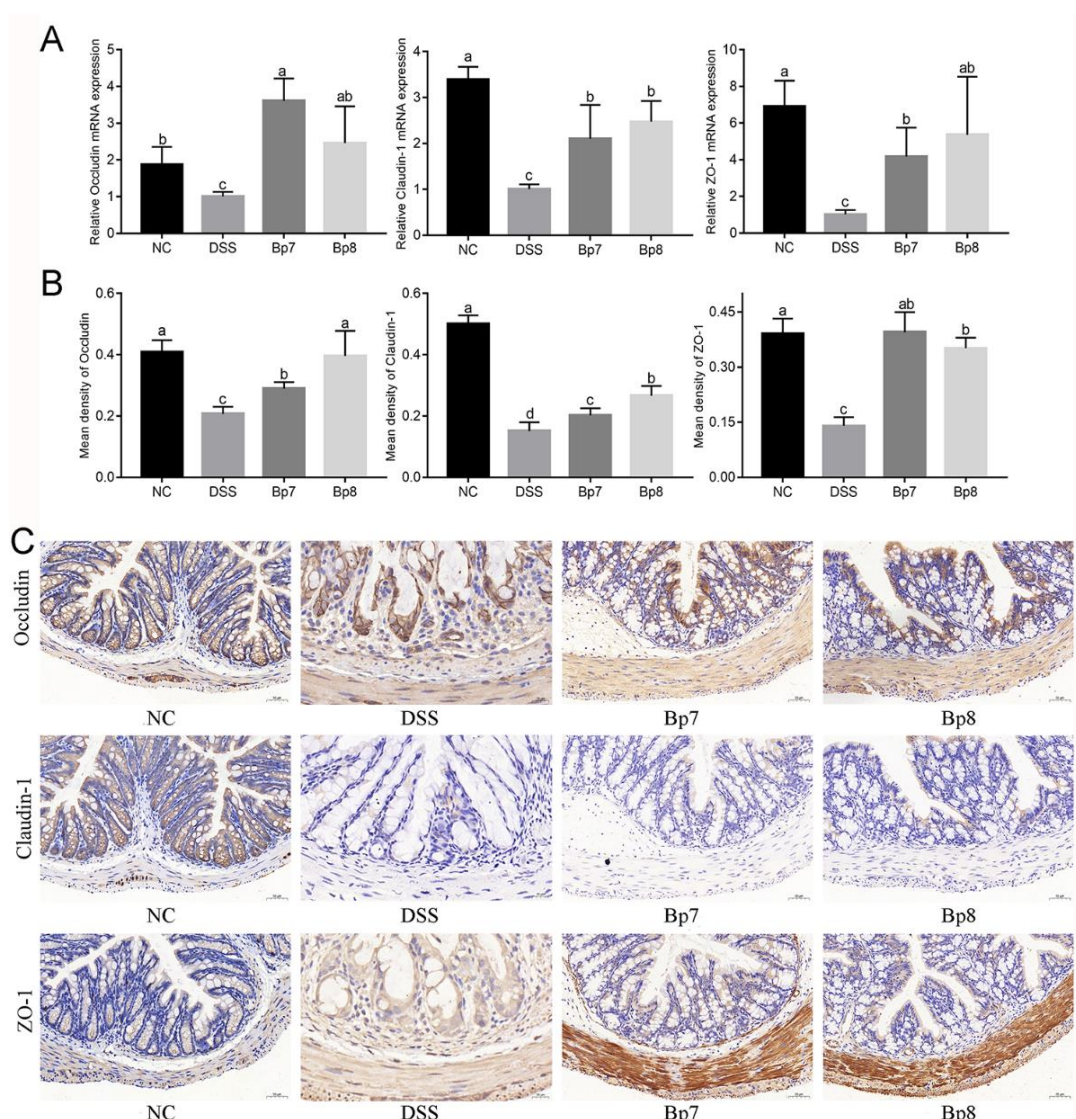
Influence of Bp7 and Bp8 intervention on the tight junction protein in the colon. The mRNA expression of colonic occludin, claudin-1, and ZO-1 (*n* = 8) (**A**); the mean density of colonic occludin, claudin-1, and ZO-1 (*n* = 8) (**B**); immunohistochemical staining for occludin, claudin-1, and ZO-1 (**C**). The different letters represent significant differences between experimental groups (*p* < 0.05).

Expression of tight junction proteins (occludin, claudin-1, and ZO-1) was modulated by *B. pseudolongum* intervention as part of the latter’s effect on the integrity of the intestinal barrier. Relative to the NC group, the transcription levels of occludin, claudin-1, and ZO-1 were remarkably decreased in the DSS group (*p* < 0.05) (Figure 5A). Nevertheless, Bp7 and Bp8 intervention obviously elevated the transcription levels of occludin, claudin-1, and ZO-1 relative to the DSS group (*p* < 0.05). These data were also confirmed by immunohistochemical analysis of the colon (Figure 5B,C), which suggested that Bp7 and Bp8 treatment effectively ameliorated damage of the intestinal barrier induced by DSS.

### 3.4. B. pseudolongum Intervention Modulates the PPARγ/STAT3 Pathway

**Figure 6:**
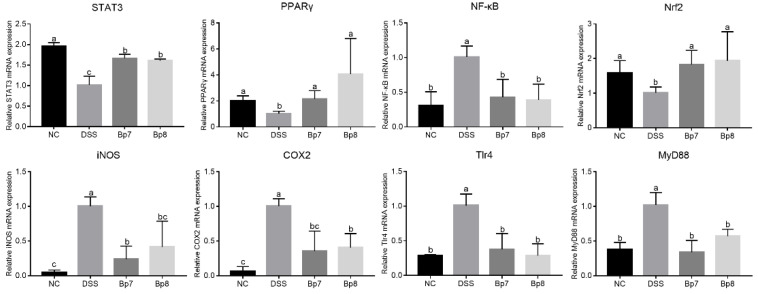
Influence of Bp7 and Bp8 intervention on the mRNA transcription of gene-related NF-κB pathway (*n* = 8). The different letters represent significant differences between experimental groups (*p* < 0.05).

To deeply analyze the influences of Bp7 and Bp8 intervention on mice with DSS treatment, the mRNA levels of genes related to colitis were measured in the NC, DSS, Bp7, and Bp8 groups (Figure 6). The transcription levels of colonic Tlr4, MyD88, NF-κB, iNOS, and COX2 were significantly elevated in mice with DSS treatment relative to those in mice without DSS treatment (*p* < 0.05), but the expression of colonic STAT3, Nrf2, and PPARγ were remarkably inhibited (*p* < 0.05). However, Bp7 and Bp8 intervention drastically down-regulated the expression of Tlr4, NF-κB, iNOS, and COX2 and drastically up-regulated the expression of STAT3, Nrf2, and PPARγ in colitis mice (*p* < 0.05).

### 3.5. B. pseudolongum Alters Intestinal Microbiota Composition

**Figure 7:**
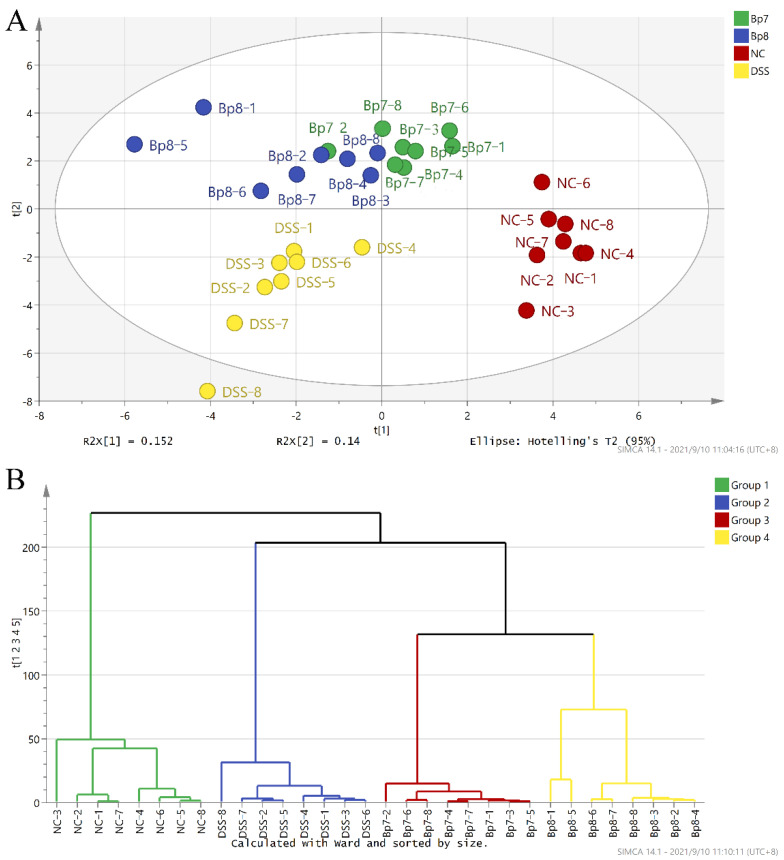
Influence of Bp7 and Bp8 intervention on the intestinal microbiota structure. PCA (**A**); HCA (**B**).

Some reports have linked the pathogenesis and progression of colitis with characteristic alterations in the intestinal microbiota structure. Thus, we examined whether *B. pseudolongum* intervention could prevent DSS-induced disturbances in intestinal microbiota composition. The results of PCA showed that the intestinal microbiota composition was altered in the DSS group compared with that in the NC group (Figure 7A). However, Bp7 and Bp8 interventions reversed these changes, especially for the Bp7 intervention. These results were further confirmed by HCA analysis (Figure 7B).

**Figure 8:**
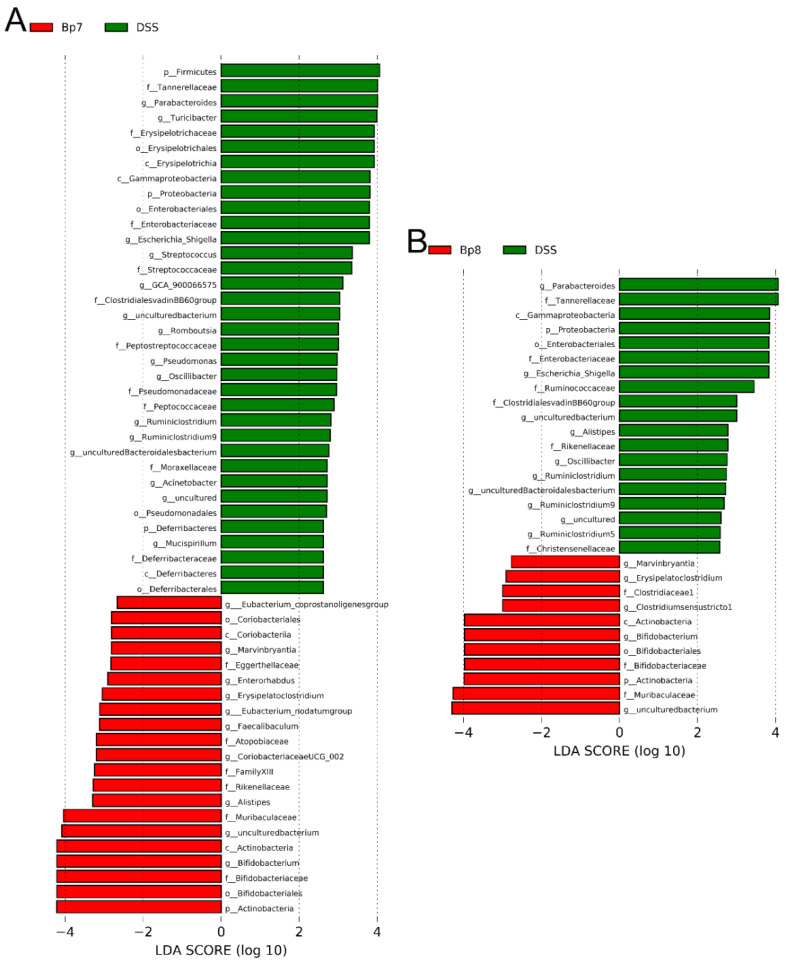
LEfSe analysis and linear discriminant analysis (LDA) score for taxa differing between Bp7 and DSS groups (**A**); Bp8 and DSS groups (**B**).

Linear discriminant analysis (LDA) was applied to determine alterations in the intestinal microbiota following Bp7 and Bp8 intervention. The relative abundances of *Lactobacillus*, *Eubacterium xylanophilum group*, *Eubacterium ventriosum group*, *Roseburia*, *Marvinbryantia,* and A2 were remarkably decreased in the DSS group in contrast to the NC group, whereas the relative abundance of *Mucispirillum*, *Acinetobacter*, *Romboutsia*, *Pseudomonas*, *Adlercreutzia*, *Escherichia_Shigella*, *Turicibacter,* and *Parabacteroides* in the DSS group were remarkably enhanced in contrast to the NC group (Figure S2). After Bp7 intervention, the relative abundance of *Eubacterium coprostanoligenes group*, *Marvinbryantia*, *Enterorhabdus*, *Erysipelatoclostridium*, *Eubacterium nodatum group*, *Faecalibaculum*, *Coriobacteriaceae* UCG 002, *Alistipes,* and *Bifidobacterium* were remarkably increased in contrast to the DSS group (Figure 8A). Similarly, Bp8 intervention led to a dramatic increase in the proportion of *Marvinbryantia*, *Erysipelatoclostridium*, *Clostridiumsensustridium,* and *Bifidobacterium* (Figure 8B). Altogether, the disturbances of intestinal microbiota in colitis mice were relieved by Bp7 and Bp8 intervention.

To explore the influence of Bp7 and Bp8 supplementation on the latent metabolic pathways of intestinal flora in DSS-treated mice, PICRUSt analysis was carried out among the NC, DSS, Bp7, and Bp8 groups. Compared with the NC group, several metabolic pathways, such as galactose metabolism, other glycan degradation, glycolysis/gluconeogenesis, amino sugar and nucleotide sugar metabolism, starch and sucrose metabolism, primary and secondary bile acid biosynthesis, and tyrosine metabolism were weakened remarkably in the DSS group (Figure S3A). However, cysteine and methionine metabolism, other glycan degradation, *n*-glycan biosynthesis, peroxisome, protein digestion and absorption, proteasome, phosphonate and phosphinate metabolism, valine, leucine and isoleucine degradation and biosynthesis, starch and sucrose metabolism, apoptosis, glycosaminoglycan degradation, streptomycin biosynthesis, pantothenate and CoA biosynthesis, glycine, serine and threonine metabolism, lipoic acid metabolism, histidine metabolism, TCA cycle, and galactose metabolism in the Bp7 group were enriched remarkably (Figure S3B). Similarly, Bp8 intervention in colitis mice resulted in an increased proportion of other glycan degradation, N-glycan biosynthesis, pentose and glucuronate interconversions, valine, leucine and isoleucine degradation and biosynthesis, RNA transport, starch and sucrose metabolism, glycosaminoglycan degradation, apoptosis, carotenoid biosynthesis, pentose phosphate pathway, protein digestion and absorption, phosphonate and phosphinate metabolism, galactose metabolism, D-arginine and D-omithine metabolism, fatty acid biosynthesis, peroxisome, beta-lactam resistance, streptomycin biosynthesis, primary and secondary bile acid biosynthesis, carbon fixation in photosynthetic organisms, and protein processing in the endoplasmic reticulum (Figure S3C).

### 3.6. Influences of B. pseudolongum on SCFA Levels in Mice with DSS Treatment

Cecal contents were analyzed to explore the influence of Bp7 or Bp8 supplementation on the levels of SCFAs (Figure S4). The cecal acetic, propionic, isobutyric, butyric, and isovaleric acid levels of mice were remarkably decreased in the DSS group compared to that in the NC group (*p* < 0.05), but there were no obvious differences in cecal valeric acid levels between the NC and DSS groups (*p* > 0.05). Notably, Bp7 intervention significantly elevated the cecal isobutyric, butyric, and isovaleric acid levels (*p* < 0.05). Bp8 treatment significantly elevated the cecal propionic, isobutyric, butyric, valeric, and isovaleric acid levels compared with those in the DSS group (*p* < 0.05).

### 3.7. The Associations between Colitis-Related Parameters and Key Microbiota

**Figure 9:**
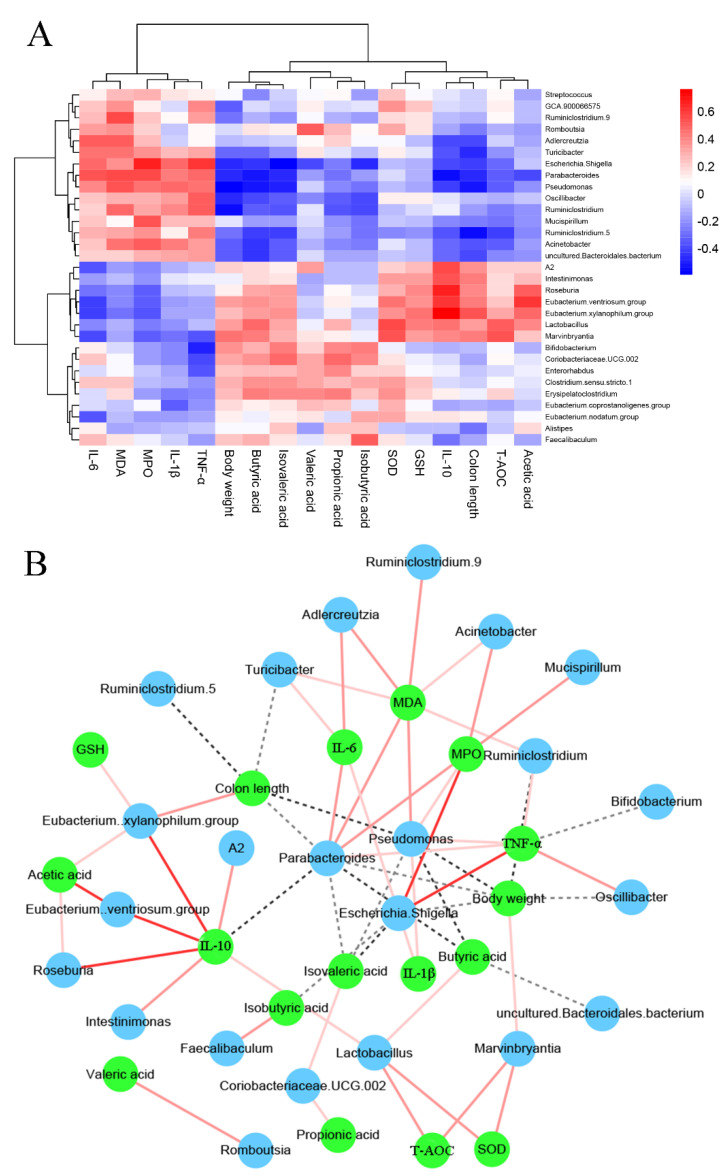
Association analysis between the key phylotypes of intestinal microbiota and colitis−related parameters. Heatmap of Spearman’s correlation (**A**) and network of Spearman’s correlation (**B**).

The potential relationship between the intestinal microbiota composition and inflammatory cytokine related to colitis was further analyzed using Spearman rank correlation (Figure 9A,B). The abundance of *Streptococcus*, GCA 900066575, *Ruminiclostridium* 9, *Romboutsia*, *Adlercreutzia*, *Turicibacter*, *Escherichia Shigella*, *Parabacteroides*, *Pseudomonas*, *Oscillibacter*, *Ruminiclostridium*, *Mucispirillum*, *Ruminiclostridium* 5, *Acinetobacter,* and uncultured *Bacteroidates* bacteria were positively related to the colonic IL-6, MDA, MPO, IL-1β, and TNF-α levels but were negatively related to body weight, colon length, SCFA levels (acetic, propionic, isobutyric, butyric, and isovaleric acids), IL-10 levels, and the activities of antioxidant enzymes (T-AOC, GSH and SOD). The abundance of A2, *Intestinimonas*, *Roseburia*, *Eubacterium ventriosum group*, *Eubacterium xylanophilum group*, *Lactobacillus*, *Marvinbryantia*, *Bifidobacterium*, *Coriobacteriaceae* UCG 002, *Enterorhabdus*, *Clostridium sensu stricto* 1, *Erysipelatoclostridium*, *Eubacterium coprostanoligenes group*, *Eubacterium nodatum group*, *Alistipes,* and *Faecalibaculum* were negatively associated with colonic IL-6, MDA, MPO, IL-1β, and TNF-α levels. Moreover, the abundance of A2, *Intestinimonas*, *Roseburia*, *Eubacterium ventriosum group*, *Eubacterium xylanophilum group*, *Lactobacillus*, and *Marvinbryantia* were positively associated with acetic acid, colon length, and antioxidant enzyme activity (T-AOC, GSH, and SOD).

## 4. Discussion

Colitis is regarded as a life-threatening disease in developed and developing countries. Conventional therapies result in tremendous economic costs and adverse side effects. Consequently, it is important to seek and develop effective and safe therapeutic agents. In this study, we showed that *B. pseudolongum* (Bp7 and Bp8) effectively mitigated intestinal barrier damage in colitis mice via regulating the PPARγ/STAT3 pathway and modulating the intestinal microbiota composition, recovering the antioxidant enzyme activities, and inhibiting pro-inflammatory cytokines.

Milk is commonly used as a protective matrix for probiotics in animal experiments. Milk exhibits favorable characteristics regarding the survival of probiotics during storage. In addition, no data were found regarding the use of milk in the treatment of colitis and irritable bowel syndrome [15,16]. Therefore, milk was used as the matrix in this study. Colitis is characterized by abnormal levels of inflammatory cytokines, especially TNF-α, IL-6, and IL-1β. TNF-α plays a crucial role in many diseases associated with inflammation and its biological properties through TNFR1 and TNFR2 interaction [17]. In addition, the proinflammatory properties of TNF-α are strongly associated with the secretion of proinflammatory cytokines, for instance, IL-6, and IL-1β [18]. IL-6 plays a vital role in the pathophysiology of inflammatory diseases by accelerating the differentiation of neutrophil and T helper 17 cells and inhibiting the differentiation of T cells [18]. Excessive secretion of IL-6 promotes the reproduction of epithelial cells and even induces colorectal cancer [18]. IL-1β is produced by infiltrating myeloid cells and is used to monitor IBD development and progression. Increases in IL-1β accelerate the growth of cancer cells by inducing immature myeloid cells. A prior study suggested that the elevation of colonic TNF-α, IL-6, and IL-1β levels in colitis mice were induced by DSS [19], which is consistent with our findings. In addition, IL-10 plays crucial immunosuppressive role in some diseases by promoting B-cell proliferation and maintaining Treg-suppressive function and then accelerating the secretion of antibodies. Our results showed that Bp7 and Bp8 intervention obviously reduced the colonic TNF-α and IL-1β levels and significantly elevated the colonic IL-10 levels, indicating that Bp7 or Bp8 effectively relieved the development of inflammation in colitis mice. However, Bp8 intervention slightly reduced the colonic IL-6 levels in colitis mice, which may be attributed to the individual differences in mice and the strain differences in *B. pseudolongum*. It is widely accepted that the accumulation of inflammatory cytokines is generally accompanied by decreased activities of antioxidant enzymes. Some studies have revealed that MPO serves as a pro-oxidative and pro-inflammatory enzyme. Moreover, its activities are strongly associated with the degree of tissue damage and progression of inflammation, and its expression directly reflects the neutrophil infiltration in colitis mice [20]. Furthermore, MDA serves as one of the vital metabolites of lipid peroxidation, and its concentration is usually applied to detect colon tissue damage in colitis [21]. Our findings suggest that Bp7 or Bp8 intervention improved DSS-induced colitis by inhibiting the activity of MPO and decreasing the concentration of MDA. Furthermore, colonic T-AOC, SOD, and GSH activities were elevated in colitis mice after Bp7 and Bp8 treatment. SOD, a key anti-oxidative enzyme, prevents the development of inflammation by transforming superoxide into oxygen and hydrogen peroxide, and the latter is degraded by catalases and peroxidases. GSH acts as the redox buffer in cells, which plays a key role in enhancing cells against oxidative damage and is strongly associated with the growth and differentiation of cells [22]. Thus, increased activities of SOD and GSH are beneficial for protecting mice against the process of colitis.

Oral administration of DSS can destroy the intestinal barrier function in mice. The intestinal barrier is a complex structure that plays an essential role in host response to gut pathogens and tolerance to the intestinal microbiota. Goblet cells are specialized cells of the intestinal epithelium, and they play a vital role in maintaining the mucosal barrier and the mucosal immune homeostasis [23]. The function of goblet cells is highly dependent on the production of Muc2, which resists attacks from pathogens as well as invasion by commensal microbiota. The deficiency of Muc2 can accelerate the process of colitis. In addition, the repair of mucosa and recovery of goblet cells are associated with the expression of MyD88 [5]. A previous study suggested that *Bifidobacterium* promotes the secretion of IL-22 by inhibiting the expression of MyD88 [24]. In the present study, Bp7 and Bp8 intervention effectively protected against DSS-induced goblet cell damage and elevated the expression of MyD88. In addition, tight junction proteins are key components located between intestinal epithelial cells, and they arrest the transmission of microbial toxins and antigens. Thus, the expression of tight junction proteins was determined at the mRNA and protein level in the present study. DSS exposure induced the reduction of the mRNA and protein levels of tight junction proteins, which is in accordance with the previous study. However, tight junction proteins were remarkably enhanced after the Bp7 and Bp8 intervention.

The mRNA levels of colonic genes related to inflammation were analyzed in order to uncover the molecular mechanisms underlying the ameliorative effects of Bp7 or Bp8 on gut inflammation in colitis mice. Bp7 and Bp8 intervention elevated the expression of STAT3 and PPARγ in colitis mice. The up-regulation of STAT3 elevated the transcription of PPARγ, which preserves intestinal homeostasis through controlling the differentiation of colon epithelial cells and regulating the function of adaptive immune effector T cells [5,25]. A recent study suggested that overexpression of PPARγ suppresses NF-κB expression. As everyone knows, NF-κB plays an essential role in regulating inflammation and host immune responses. Overexpression of NF-κB induces the hyperactivation of inflammatory response and promotes the release of proinflammatory cytokines (containing TNF-α, IL-6, and IL-1β), even facilitating the occurrence of colitis. Conversely, blocking NF-κB transcriptional activity suppressed the mRNA transcription of iNOS and COX2. iNOS plays a vital role in the pathogenesis of many inflammatory syndromes, such as irritable bowel syndrome and rheumatoid arthritis [26]. Inhibiting iNOS transcription is an effective strategy for preventing the pathogenesis and development of colitis. COX2 is a vital indicator of colonic mucosal inflammation, and its transcription is significantly increased in patients with IBD. A previous study reported that elevated COX2 expression is closely associated with the IL-1 signaling pathway [27]. Our results suggest that down-regulation of COX2 and iNOS promotes gut barrier integrity, protecting against DSS-induced colitis after Bp7 and Bp8 intervention. Thus, Bp7 and Bp8 ameliorate colitis by activating the PPARγ/STAT3 pathway. 

Alterations in intestinal microbiota composition are closely associated with the development of several diseases, for instance, colitis, obesity, and diabetes. Among those, colitis is regarded as a result of an inappropriate response of the mucosal immune system induced by the intestinal microbiota, characterized by the increase of potentially harmful bacteria and the decrease of potentially beneficial bacteria. A previous study showed that the changes in intestinal microbiota structure exacerbate low-grade mucosal inflammation [28]. It is widely believed that dietary DSS disturbs the intestinal microbiota composition, consistent with the results of this study. Our data showed that DSS treatment in mice remarkably reduced the relative abundance of *Lactobacillus*, *Eubacterium xylanophilum group*, *Eubacterium ventriosum group*, *Roseburia*, *Marvinbryantia,* and A2 compared with that in mice without DSS treatment. Among those, *Lactobacillus*, *Roseburia,* and *Marvinbryantia* belonging to the Firmicutes phylum and are thought to be probiotic [29]. *Lactobacillus* is a gram-positive bacterium that relieves the symptoms of inflammation by regulating the NF-κB pathway. *Lactobacillus* intervention maintains intestinal mucosal integrity, elevates the secretion of tight junction proteins, and inhibits the expression of immune factors [30]. *Roseburia* and *Marvinbryantia* are butyrate-producing bacteria, and their abundance is strongly related to the concentration of butyrate in the gastrointestinal tract [31]. Butyrate ameliorates intestinal barrier integrity and accelerates cell motility by suppressing the activity of histone deacetylase and promoting the expression of the actin-binding protein synaptopodin (SYNPO) [32]. Our results showed that DSS treatment in mice significantly reduced the cecal butyric acid concentration when in contrast to that in mice without DSS treatment, which is consistent with the previous report [33]. In contrast, *Mucispirillum* and *Pseudomonas* are considered pathobionts because they can cause several diseases under certain conditions, especially IBD [34]. *Acinetobacter* are rare in the intestinal microbiota of healthy individuals, and they stimulate proinflammatory responses in monocolonized mice [35]. These specific bacterial pathogens can bind to host-derived proinflammatory cytokines. In addition, a previous study revealed that a high-energy diet could promote the development of colitis because it enhanced the concentration of cholesterol in the intestinal tract. The enriched abundance of the *Eubacterium coprostanoligenes* group leads to increased excretion of 7α-dehydroxylase, which in turn elevates the conversion of intestinal cholesterol to bile acid [36]. *Enterorhabdus* can hydrolyze primary bile acids, which facilitates the excretion of bile acid [37]. *Marvinbryantia* and *Coriobacteriaceae* UCG 002 have recently attracted growing interest due to their anti-inflammatory role. High abundance of *Marvinbryantia* and *Coriobacteriaceae* UCG 002 results in the elevation of SCFA concentration, which suppresses the differentiation of naive CD4+ T cells and thus impacts the differentiation of helper T (Th) cells [31]. High concentrations of secondary bile acid and SCFAs upregulate the expression of P-glycoprotein, which inhibits overactive inflammation and maintains intestinal homeostasis. Our data suggest that the abundance of *Eubacterium coprostanoligenes group*, *Marvinbryantia*, *Enterorhabdus*, *Faecalibaculum*, *Coriobacteriaceae* UCG 002, *Alistipes,* and *Bifidobacterium* in colitis mice significantly increased after *B. pseudolongum* intervention. Notably, the association between *Alistipes* and the host health is still disputed. A recent report suggested that *Alistipes* could produce succinic acid, which helps to maintain intestinal homeostasis by accelerating cell differentiation, promoting tight junction assembly, and increasing the expression of proglucagon in intestinal L cells [38]. In addition, *Faecalibaculum*, a kind of lactic acid producer, is known to be strongly negatively related to *inflammatory* cytokines (IL-6 and TNF-α) [39]. Taken together, our results clearly elaborate that *B. pseudolongum* (Bp7 and Bp8) intervention regulates the intestinal microbiota structure, and its collective effects suppress intestinal inflammation.

## 5. Conclusions

Bp7 and Bp8 intervention ameliorated the intestinal barrier function by activating the PPARγ/STAT3 pathways and regulating the intestinal microbiota composition. Our data reveal the essential role of *B. pseudolongum* (Bp7 and Bp8) in the pathogenesis and development of colitis. However, additional metabolomic and metagenomic studies are needed to further explore the specific metabolites derived from *B. pseudolongum* that are involved in ameliorating the damage to the intestinal barrier function.

## Data Availability

The data presented in this study are available on request from the corresponding author. The data are not publicly available due to privacy.

## Supplementary Materials

The following supporting materials can be downloaded at: https://www.mdpi.com/article/10.3390/foods11111551/s1. Table S1. Calculated disease activity index (DAI) score; Table S2. Primer sequences for quantitative real-time PCR; Figure S1. The flow chart of animal experiment. Figure S2. LEfSe identified the gut microbiota phylotypes with a statistically significant difference in abundance between NC and DSS groups; Figure S3. Predicted metabolic functions for the altered genes of intestinal microbiota. Metabolic pathways of NC vs. DSS (A), Bp7 vs. DSS (B) and Bp8 vs. DSS (C); Figure S4. Influence of Bp7 and Bp8 intervention on the cecal SCFA concentrations.
