# Supplementary material for: Protective Effects of a Novel Probiotic Bifidobacterium pseudolongum on the Intestinal Barrier of Colitis Mice via Modulating the Pparγ/STAT3 Pathway and Intestinal Microbiota
Source: Foods. 2022 May 25;11(11):1551. doi: 10.3390/foods11111551 (PMC9180506; doi:10.3390/foods11111551)
Supplement: Supplementary file 1 [file foods-11-01551-s001.zip › foods-1710836-supplementary.pdf]

Supplementary Materials

Supplementary Material 1：

Table S1. Calculated disease activity index (DAI) score.

Score	Weight loss (%)	Stool consistency	Stool bleeding
0	no loss	normal	occult blood test-negative
1	1.0-5.0	soft but formed	occult blood test-positive
2	5.0-10.0	soft	slight
3	10.0-15.0	slight diarrhea	blood traces in stool
4	more than 15.0	watery diarrhea	gross rectal bleeding

Supplementary Material 2:

Table S2. Primer sequences for quantitative real-time PCR

Gene	Forward primer (5′−3′)	Reverse primer (5′−3′)
STAT3	GAGGAGGCATTCGGAAAG	TCGTTGGTGTCACACAGAT
PPARγ	TGGGTGAAACTCTGGGAGAT	CCATAGTGGAAGCCTGATGC
NF-κB	CGCCCCTTATCGACCACC	CCTTCTCCCAAGAGTCGTCCA
COX2	GAAGTCTTTGGTCTGGTGCCTG	GTCTGCTGGTTTGGAATAGTTG
iNOS	CTTGGAGCGAGTTGTGGATTGTC	TAGGTGAGGGCTTGGCTGAGT
Nrf2	CCTCCGCTGCCATCAGTCAGT	TCGGCTGGGACTCGTGTTCA
MyD88	GCATGGTGGTGGTTGTTTCTG	GAATCAGTCGCTTCTGTTGG
Occludin	CCGGCCGCCAAGGTTC	GCTGATGTCACTGGTCACCTA
Claudin-1	GGCTTCTCTGGGATGGATCG	CCCCAGCAGGATGCCAATTA
ZO-1	GCCTTGAACTTTGACCTCTGC	GAAATCGTGCTGATGTGCCA
GAPDH	AGGTCGGTGTGAACGGATTTG	GGGGTCGTTGATGGCAACA

Supplementary Material 3:

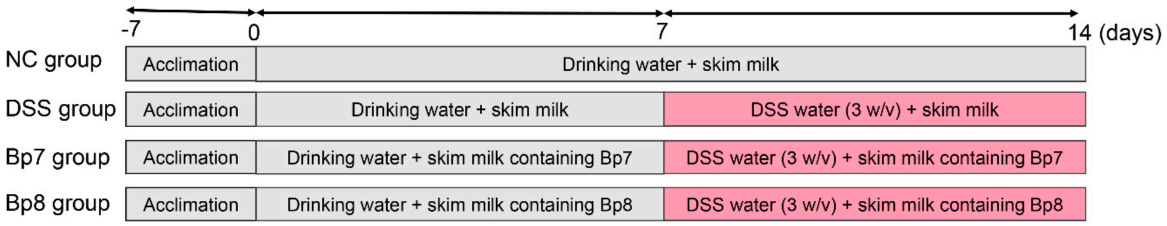

Figure S1 The flow chart of animal experiment.

Supplementary Material 4:

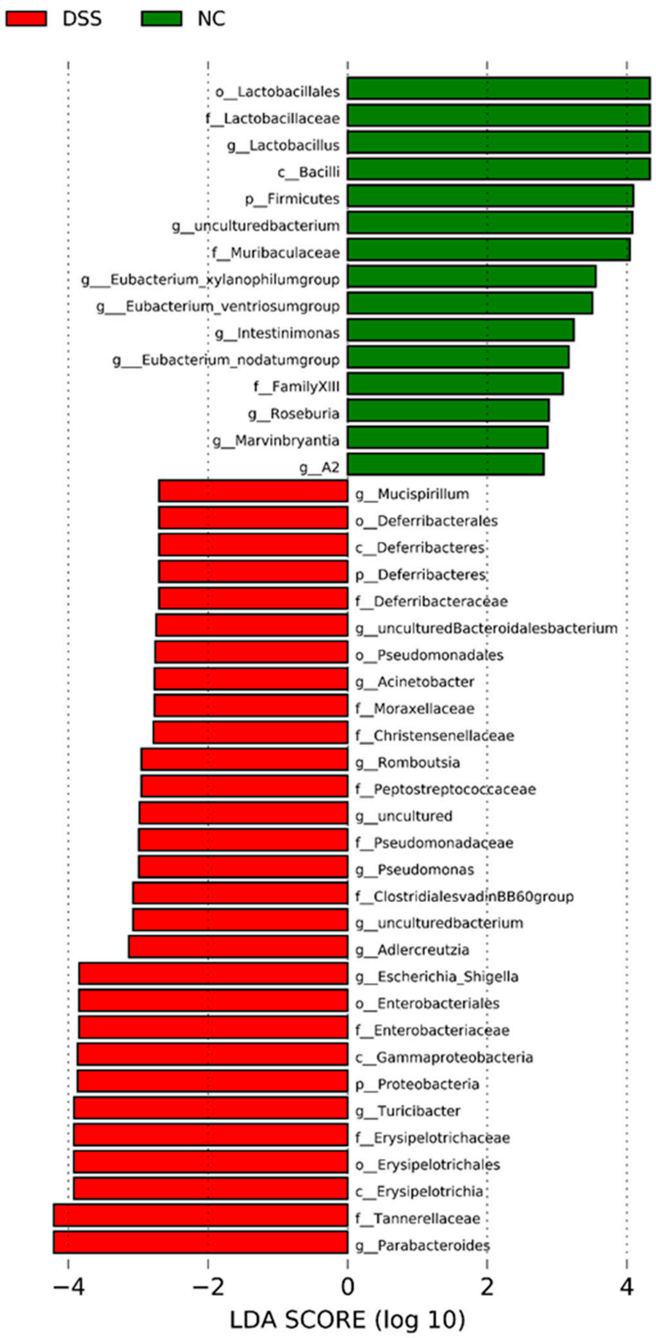

Figure S2. LEfSe identified the gut microbiota phylotypes with a statistically significant difference in abundance between NC and DSS groups.

Supplementary Material 5:

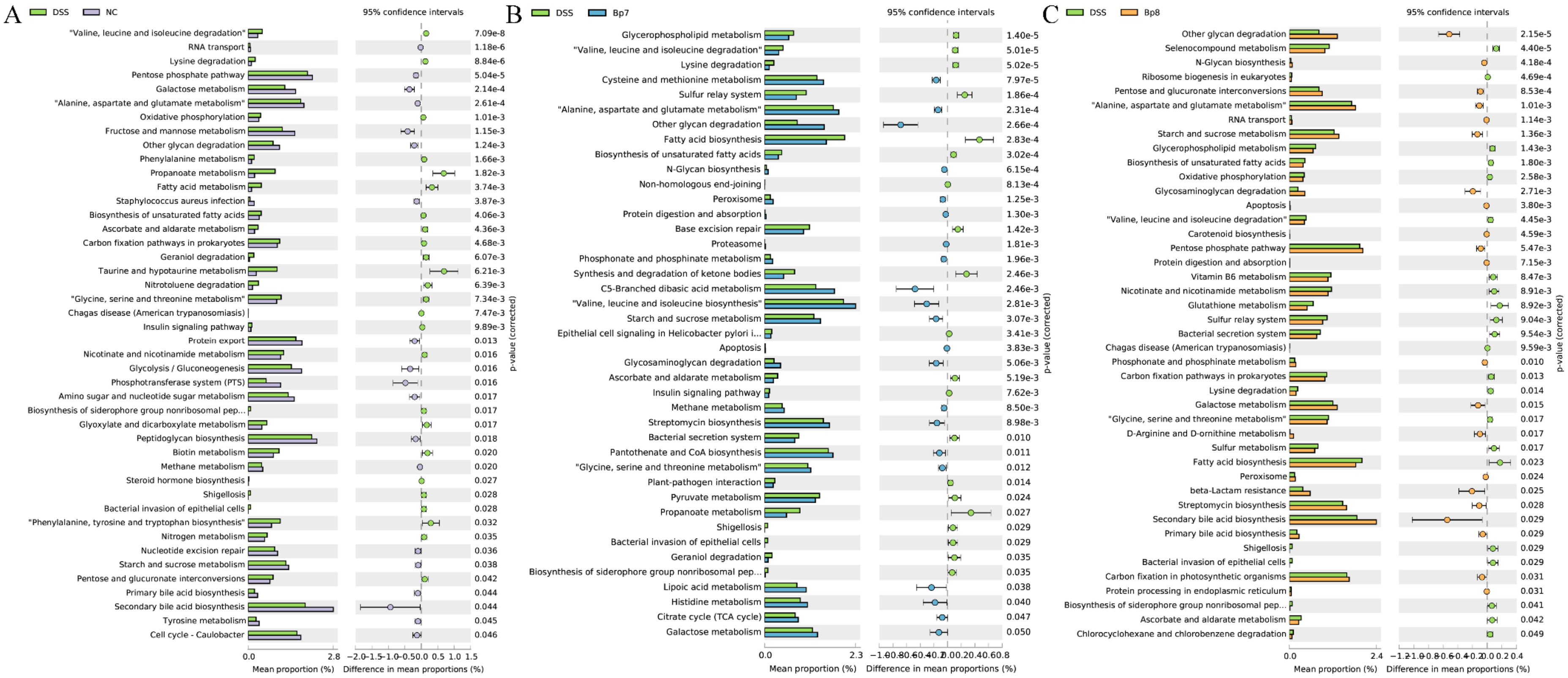

Figure S3. Predicted metabolic functions for the altered genes of intestinal microbiota. Metabolic pathways of NC vs DSS (A), Bp7 vs DSS (B) and Bp8 vs DSS (C).

Supplementary Material 6:

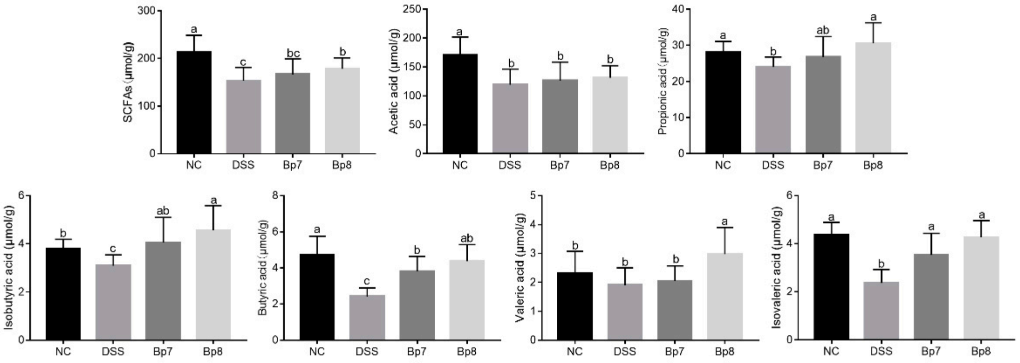

Figure S4. Influence of Bp7 and Bp8 intervention on the cecal SCFAs concentrations. Values are showed as the mean ± SD (n = 8). The different letters represent significant differences between different experimental groups ($P < 0.05$).
